# Immunotherapy with immune checkpoint inhibitors and predictive biomarkers in malignant mesothelioma: Work still in progress

**DOI:** 10.3389/fimmu.2023.1121557

**Published:** 2023-01-27

**Authors:** Matteo Perrino, Fabio De Vincenzo, Nadia Cordua, Federica Borea, Marta Aliprandi, Armando Santoro, Paolo Andrea Zucali

**Affiliations:** ^1^ Department of Oncology, Istituto di Ricovero e Cura a Carattere Scientifico (IRCCS) Humanitas Research Hospital, Milan, Italy; ^2^ Department of Biomedical Sciences, Humanitas University, Milan, Italy

**Keywords:** immunotherapy, immune checkpoint inhibitors, biomarkers, predictive of response, malignant mesothelioma

## Abstract

Malignant mesothelioma (MM) is a rare and aggressive neoplasm, usually associated with a poor prognosis (5 years survival rate <10%). For unresectable disease, platinum and pemetrexed chemotherapy has been the only standard of care in first line for more than two decades, while no standard treatments have been approved in subsequent lines. Recently, immunotherapy has revolutionized the therapeutic landscape of MM. In fact, the combination of ipilimumab plus nivolumab has been approved in first line setting. Moreover, immune checkpoint inhibitors (ICIs) showed promising results also in second-third line setting after platinum-based chemotherapy. Unfortunately, approximately 20% of patients are primary refractory to ICIs and there is an urgent need for reliable biomarkers to improve patient’s selection. Several biological and molecular features have been studied for this goal. In particular, histological subtype (recognized as prognostic factor for MM and predictive factor for chemotherapy response), programmed death ligand 1 (PD-L1) expression, and tumor mutational burden (widely hypothesized as predictive biomarkers for ICIs in several solid tumors) have been evaluated, but with unconclusive results. On the other hand, the deep analysis of tumor infiltrating microenvironment and the improvement in genomic profiling techniques has led to a better knowledge of several mechanisms underlying the MM biology and a greater or poorer immune activation. Consequentially, several potential biomarkers predictive of response to immunotherapy in patients with MM have been identified, also if all these elements need to be further investigated and prospectively validated.

In this paper, the main evidences about clinical efficacy of ICIs in MM and the literature data about the most promising predictive biomarkers to immunotherapy are reviewed.

## Introduction

Malignant mesothelioma (MM) is a rare and aggressive neoplasm originating from the mesothelial lining of the pleural cavity (1). Its annual incidence is globally increasing and it is closely related to asbestos exposure (accounting 80% of cases), with a long latency of almost 40 years between exposure and the disease onset. In general, the prognosis of MM is poor, with a median survival not exceeding 14 months and with a 5 years survival rate less than 10%. In Europe, according to the differences in terms of asbestos exposure, MM is more frequent in males (1.7/1000) than in females (0.4/1000). At diagnosis, median age is 70 years old in western countries. According to the World Health Organization (WHO) 2021 classification, MM is categorized in three main histological subtypes: epithelioid (50-70% of cases), characterized by a better prognosis, sarcomatoid (10-20% of cases), more aggressive and typically chemo-resistent, and biphasic, with features of both the previous (2–4).

The therapeutic landscape of mesothelioma is changing. In first line setting, platinum and pemetrexed chemotherapy has been the standard of care for unresectable disease since 2004 and no other treatments have been approved in the second- and third-line setting (1, 5). However, the immunotherapy revolution has improved the survival outcomes of patients with a broad range of cancers, including mesothelioma. In fact, the combination of ipilimumab plus nivolumab was recently approved by the US Food and Drug Administration (FDA) and the European Medicines Agency (EMA) based on the results of the randomized phase III CheckMate 743 trial (6). In this study, nivolumab plus ipilimumab significantly improved overall survival (OS) versus platinum-pemetrexed chemotherapy in unresectable chemo-naive MM patients. The 3-year updates of efficacy and safety analyses showed, after a minimum follow-up of 35.5 months, that immunotherapy with ipilimumab plus nivolumab continued to provide OS benefit over chemotherapy (HR 0.75) and 28% of patients had an ongoing response at 3 years in the immunotherapy arm (7). In second line setting, nivolumab achieved a statistically significant improvement of both progression-free survival (PFS) and OS compared to placebo in pre-treated MM patients in the randomized, phase III, CONFIRM trial (8). Lastly, several ongoing phase III trials should provide robust evidence for any benefits from combining immunotherapy with chemotherapy in the first-line setting (1).

Despite these exciting results, approximately 20% of patients are primary refractory to immunotherapy (6). Unfortunately, in clinical setting there is not yet the availability of predictive biomarkers able to guide the selection of patients really benefiting from immunotherapy. Moreover, compared with other malignancies, progress in MM biomarker research is limited.

In this paper, the main evidences about clinical efficacy of immuno-checkpoint inhibitors (ICIs) in patients with MM and the literature data regarding the biomarkers potentially predictive of response to immunotherapy are reviewed.

## Immune checkpoint inhibitors in malignant mesothelioma

Tumor-infiltrating lymphocytes (TILs), macrophages, and natural killer (NK) cells usually infiltrate the tumor tissue of mesothelioma. Epithelioid mesothelioma presents an increased stromal infiltration by TILs and helper-1-polarized T cells, whereas sarcomatoid mesothelioma is infiltrated by TILs with a high CD8+ population and a low CD4+ population and presents an increased expression of immune checkpoint programmed death ligand-1 (PD-L1). Moreover, an immunosuppressive environment is promoted through M2 polarized macrophages and regulatory T (Treg) cells. Starting from this scenario, an effort for the identification of therapies modulating the immune system, including dendritic cell (DC) therapy, chimeric antigen receptor (CAR) T-cell therapy, cancer vaccines, and checkpoint inhibitors, is ongoing. In the last decade, monoclonal antibodies directed against cytotoxic T lymphocyte antigen 4 (CTLA4) or programmed cell death (PD-1) or its ligand PD-L1 have received regulatory approval across the globe, alone or in combination with chemotherapy for the treatment of tumors, including thoracic cancer such as mesothelioma. In mesothelioma, the main evidence regards front-line and salvage settings, while neoadjuvant/adjuvant and multimodality treatment trials are still ongoing. The main results of ICIs are presented below and summarized in **Table 1**.

**Table 1:** Main trials of ICIs in malignant mesothelioma.

Name study		Phase		N pts		Drugs		Line		ORR (%)		mPFS (months)	mOS (months)
Monotherapy													
MESO-TREM (2008) (9)		II		29		Tremelimumab		2		7.0		6.2	10.7
DETERMINE (2017) (10)		IIb		571		Tremelimumab *vs* placebo (R2:1)		2-3		4.5 *vs* 1.1		2.8 *vs* 2.7	7.7 *vs* 7.3
KEYNOTE-028 (2017) (11)		Ib		35		Pembrolizumab		2-5		20.0		5.4	18.0
KEYNOTE-158 (2021) (12)		II		118		Pembrolizumab		2-5		8.0		2.1	10.0
PROMISE-MESO (2020) (13)		III		144		Pembrolizumab *vs* CHT		2		22.0 *vs* 6.0 (P = 0.004)		2.5 *vs* 3.4 (P = 0.76)	10.7 *vs* 12.4 (P = 0.85)
NIVO MES (2018) (14)		II		34		Nivolumab		2-3		24.0		2.6	11.8
MERIT (2019) (15)		II		34		Nivolumab		2-3		29.0		6.1	17.3
CONFIRM (2021) (8)		III		332		Nivolumab *vs* placebo (R2:1)		2		11.0 *vs* 1.0 (p=0.00086)		3.0 *vs* 1.8 (p=0.0012)	10.2 *vs* 6.9 (p=0.0090)
JAVELIN (2019) (16)		Ib		53		Avelumab		2-5		9.0		4.1	10.7
Combination therapy													
NIBIT-MESO 1 (2018) (17)		II		40		Tremelimumab + Durvalumab		2-3		28.0		5.7	16.6
INITIATE (2019) (18)		II		34		Nivolumab + Ipilimumab		2-5		29.0		6.2	NR
MAPS2 (2019) (19)		II		125		Nivolumab +/− Ipilimumab		2-5		Nivo arm 19.0 Ipi-Nivo arm 28.0		Nivo arm 4.0 Ipi-Nivo arm 5.6	Nivo arm 11.9 Ipi-Nivo arm 15.9
CHECKMATE 743 (2021) (6)		III		605		Nivolumab + Ipilimumab *vs* CHT		1		40 *vs* 44		6.8 *vs* 7.2	18.1 *vs* 14.1 (p=0.0020)
DREAM (2020) (20)		II		54		Durvalumab + CDDP + pemetrexed		1		48		6.9	NR
PrE0505 (2020) (21)		II		55		Durvalumab + CDDP + pemetrexed		1		56.4		6.7	20.4

PTS, patients; ORR, overall response rate; mPFS, median progression free survival. mOS, median overall survival; CHT, chemotherapy.

### Immune checkpoint inhibitors as salvage therapy

The CTLA4 inhibitor tremelimumab was the first immune checkpoint inhibitor assessed in mesothelioma. In the phase II MESO-TREM 2008 study, tremelimumab administered at the dose of 15 mg/kg every 90 days in relapsed disease setting, showed a low but durable activity with an overall response rate (ORR) of 7% (2 of 29 patients) lasting up to 18 months (9). A more intensive schedule of intravenous tremelimumab (10 mg/kg 4-weekly for seven doses, then every 12 weeks until treatment discontinuation) was compared to placebo in the randomized, double blind, phase 2b DETERMINE study. The study enrolled 571 patients, with previously treated MM, randomized 2:1 to tremelimumab or placebo arm. The median age was 66 years and 83% of patients presented epithelioid histology. The primary endpoint of the study was not reached: no statistically significant difference in terms of OS was observed between the two arms, with median OS of 7.7 months in the tremelimumab arm and 7.3 months in the placebo arm (HR 0.92, p 0.41). The ORR observed was only 4.5% and patients with sarcomatoid subtype seemed to benefit better from the CTLA4 inhibitor than patients with epithelioid subtype (10). Therefore, tremelimumab as monotherapy is not indicated for second/third-line therapy in MM.

Pembrolizumab was the first PD-1 inhibitor studied in patients with MM. KEYNOTE-28 was a single arm, phase 1b, multicohort basket trial that treated patients with PD-L1 positive (defined as ≥1% expression in the tumor cells) tumors (11). Thirty-five patients with pleural mesothelioma, who had failed to standard therapy, received pembrolizumab 10 mg/kg every two weeks up to 2 years. The median age was 65 years and 72% of patients had epithelioid histology. Primary endpoints were safety, tolerability and ORR. Five patients (20%) achieved objective response whereas 13 patients (52%) had stable disease with a median duration of response of 12 months. There was no treatment related mortality and there were no discontinuations of therapy attributable to treatment related adverse events.

In the single arm, open label, phase 2 KEYNOTE-158 trial, 118 patients with previously treated mesothelioma, received pembrolizumab 200 mg every 21 days for up to 35 cycles. Primary endpoint was ORR. Ten out of 118 patients (8%) had an objective response. Median duration of response was 14.3 months and 60% of objective response were ongoing at 12 months. Stratifying for PD-L1 expression, objective responses were observed in six out of 77 patients (8%) with PD-L1 positive tumor (median duration of response: 17.7 months) and in four out of 31 patients (13%) with PD-L1 negative tumor (median duration of response: 10.2 months). Median OS and the median PFS were 10 months (95% CI 7.6–13.4) and 2.1 months (95% CI 2.1–3.9), respectively. In conclusion, pembrolizumab showed durable anti-tumor activity in patients with advanced MM, regardless of PD-L1 status (12). In the phase 3 PROMISE-MESO trial, a total of 144 patients who had progressed after previous platinum-based chemotherapy and regardless of PD-L1 expression, were randomized 1:1 to pembrolizumab 200 mg every three weeks or physician’s choice of chemotherapy gemcitabine at 1000 mg/m2 (days 1 and 8) every 3 weeks or vinorelbine at 30 mg/m2 IV (days 1 and 8) until progression. The primary endpoint was PFS. The median age was 70 years, with almost 90% having epithelioid histology. Although ORR with pembrolizumab was 22% compared to 6% with chemotherapy, the study did not show a statistically significant improvement in median PFS or in median OS even stratifying by PD-L1 expression status. Median PFS was 2.5 months in the pembrolizumab group versus 3.4 months in the chemotherapy group (HR 1.06; p=0.76). Median OS was 10.7 months in the pembrolizumab arm versus 12.4 months in the chemotherapy arm (HR 1.12; p=0.59) (13).

Nivolumab has been evaluated as monotherapy in two phase 2 trials. The Dutch study NivoMes was a single-center, single arm study of 34 patients enrolled to receive nivolumab (3 mg/kg) every two weeks for up to 12 months. The primary endpoint was disease control rate (DCR) assessed at 12 weeks ≥40%. The study met its primary endpoint with DCR at 12 weeks of 47%. The median PFS and median OS were 2.6 months and 11.8 months, respectively. Half of the patients with stable disease (n=4), achieved disease stability for more than 6 months. The safety profile included one treatment-related death from pneumonitis. Responses by PD-L1 status showed that PD-L1 expression did not correlate with survival outcomes (14). The MERIT trial was an open label, single arm, phase 2 study of 34 patients enrolled to receive nivolumab 240 mg every two weeks until progression disease or unacceptable toxicity. The primary endpoint was ORR. Ten out of 34 patients (29%) achieved an objective response. The median PFS and OS were 6.1 months and 17.3 months, respectively. In this trial, PD-L1 expression (≥1% *vs <*1%) had an impact in terms of ORR (40% versus 8%), PFS (7.2 months versus 2.9 months), and OS (17.3 months versus 11.6 months) even if not statistically significant. Based on these results, the Japanese MERIT trial was the world’s first study to obtain regulatory approval for a checkpoint inhibitor in August 2018 (15). The phase 3 CONFIRM trial demonstrated that nivolumab improves PFS and OS over placebo in 332 patients randomized 2:1 to nivolumab at dose of 240 mg IV every 14 days or placebo until disease progression or a maximum of 12 months (8). Of note, 57% of patients were treated in the 3^rd^ line setting. The co-primary endpoints PFS and OS were met: Nivolumab achieved a statistically significant improvement in terms of both mPFS (HR: 0.67; p=0.0012) and mOS (HR: 0.69; p=0.0090). If the PD-L1 expression (≥1%) was not predictive for either PFS or OS, a statistically significant improvement in terms of PFS and OS was reported in the subgroup analysis in patients with epithelioid histology but not in non-epithelioid patients. These data justify using an anti-PD-1 inhibitor in MM patients after failure with platinum-pemetrexed-based chemotherapy.

The safety and efficacy of avelumab have been investigated in the large, multicohort phase 1b JAVELIN study. Fifty-three patients with pretreated mesothelioma received avelumab 10 mg/kg every two weeks until progression disease or unacceptable toxicity. The confirmed ORR was 9% (5 patients: 95% CI, 3.1%-20.7%), with complete response in 1 patient and partial response in 4 patients. The median PFS and OS were 4.1 months and 10.7 months, respectively. According to PD-L1 tumor expression (positive PD-L1≥5% versus negative PD-L1<5%), a higher ORR (14.3% versus 8.0%) and a longer PFS (17.1 weeks versus 7.4 weeks) were observed in the PD-L1-positive group (16).

Trials investigating combinations of ICIs targeting either PD-1 or PD-L1 with anti-CTLA4 antibodies in a salvage setting emerged almost in parallel with those testing ICIs as monotherapy.

The combination of the anti-CTLA-4 tremelimumab (1 mg/kg) and the anti-PD-L1 durvalumab (20 mg/kg) administered every 4 weeks for up to 4 doses followed by maintenance durvalumab alone was evaluated in the open-label, single-arm, phase 2 NIBIT-MESO-1 trial. In total, 40 patients with MM (28 pre-treated and 12 treatment-naïve patients) were enrolled. The primary endpoint of this study (immune-related ORR ≥25%) was met: the ORR was 28% in all populations and 33% in the treatment naïve patients. The median duration of response was 16.1 months, but the tumor PD-L1 expression was not associated with better ORR or longer survival outcomes. Despite positive results, the small sample size of this trial does not justify the use of these drugs in clinical practice (17).

The single-arm phase 2 INITIATE trial studied nivolumab (240 mg every 2 weeks) combined with ipilimumab (1 mg/kg every 6 weeks up to 4 doses) in 34 patients with MM relapsed after platinum-based therapy (18). The primary endpoint was met because a 12-weeks DCR of 67% was observed. Response to therapy resulted higher in patients with PD-L1 expression (≥1%) compared with patients with negative tumors (47% versus 16%).

The MAPS2 trial, a non-comparative, randomized phase II study, enrolled 125 MM patients to receive Nivolumab alone (3 mg/kg every 2 weeks) or combined with ipilimumab (1 mg/kg every 6 weeks) after platinum-based chemotherapy (19). The primary endpoint of this trial was the DCR at 12 weeks (at least 40% of patients with disease control) and it was reached in both arms (nivolumab arm: 44%; nivolumab-ipilimumab arm: 50%). The combination nivolumab/ipilimumab showed a higher ORR (28% versus 19%), a longer mPFS (5.6 months versus 4.0 months) and mOS (15.9 months versus 11.9 months), and a higher grade 3-4 treatment-related adverse events (AEs) incidence (26% versus 14%) compared to nivolumab alone. In the exploratory analysis, PD-L1 expression (≥1%) resulted correlated with higher ORR, but not with 12-week DCR. Despite the lack of FDA approval due to the absence of randomized comparisons with other treatments, the National Comprehensive Cancer Network Clinical (NCCN) Practice Guidelines in Oncology recommend nivolumab with or without ipilimumab as a preferred treatment option (Category 2A) in second-line or later settings.

In summary, the results of the phase II MAPS2 and MERIT trials and the results of the randomized phase III CONFIRM trial support using an anti-PD-1 inhibitor (in particular nivolumab) as monotherapy in patients progressing during or after platinum-pemetrexed-based chemotherapy, representing a new therapeutic horizon in second-line setting for MM.

### Immune checkpoint inhibitors in first line setting

The CheckMate-743 trial is the first phase III study demonstrating an OS improvement achieved by immunotherapy with the combination nivolumab/ipilimumab compared to standard platinum-pemetrexed chemotherapy in first-line setting in patients with unresectable MM (6). Overall, 605 patients not selected for PD-L1 expression were randomized to receive ipilimumab (1 mg/kg every 6 weeks) and nivolumab (3 mg/kg every 2 weeks) for up to 2 years versus standard platinum-pemetrexed therapy. The primary endpoint OS was met. In fact, patients treated with immunotherapy achieved a statistically significant longer OS (18.1 months versus 14.1 months; HR: 0.74; p=0.0020). Moreover, a statistically significant advantage in OS was described despite histology (mOS 18.7 months in epithelioid histology and 18.1 months in non-epithelioid one), with a greater benefit versus chemotherapy in PD-L1 (≥1%) tumor positive expression (mOS 18.0 versus 13.3 months, HR 0.69) or in non-epithelioid tumors (mOS 18.1 versus 8.8 months, HR 0.46). The ORR resulted comparable (immunotherapy arm: 40%; chemotherapy arm: 43%) whereas the duration of response (DOR) was longer in the immunotherapy arm (median 11.0 months versus 6.7 months). Both arms achieved similar mPFS (immunotherapy arm: 6.8 months; chemotherapy arm: 7.2 months; HR 1.00) whereas the incidence of grade 3-4 treatment-related AEs resulted comparable (immunotherapy arm: 30%; chemotherapy arm: 32%). Therefore, the combination ipilimumab/nivolumab was approved as first-line therapy for patients with unresectable MM either by FDA and by EMA. The 3-year updates of efficacy and safety analyses confirmed the advantage of immunotherapy with ipilimumab/nivolumab compared to chemotherapy in terms of OS (HR 0.75) (7). Of note, 28% of patients in the immunotherapy arm had an ongoing response at 3 years.

With the aim to increase the efficacy of immunotherapy in patients with mesothelioma, ICIs were also evaluated in combination with chemotherapy, tyrosine kinase inhibitors (TKIs) and other therapeutic strategies.

The single-arm, phase II DREAM trial studied the efficacy of chemo-immunotherapy by administering durvalumab (1125 mg), cisplatin (75 mg/m2), and pemetrexed (500 mg/m2) every 3 weeks for up to 6 cycles and then durvalumab as maintenance therapy up to 12 months (20). The 6-week PFS in the intention to treat population was the primary endpoint of this study. A total of 54 MM patients unselected for PD-L1 expression were enrolled. After a median follow-up of 28.2 months, 57% of patients were progression-free and alive at 6 months. The mPFS was 6.9 months, and the ORR was 48%. The PD-L1 expression did not correlate with treatment outcomes.

The single-arm, phase II PrE0505 trial is evaluating first-line immunotherapy with durvalumab and platinum-based chemotherapy and then durvalumab alone as maintenance treatment (21). The primary endpoint was the OS compared to historical control with cisplatin-pemetrexed chemotherapy. A total of 55 MM patients were enrolled. The primary endpoint of this study was met: the combination of chemotherapy with durvalumab achieved a median OS of 20.4 months compared to 12.1 months with historical control and the estimated 12-months OS rate was 70.4%. The mPFS was 6.7 months whereas the ORR was 56.4%. The genomic and immune cell repertoire analyses showed that: a higher immunogenic mutations burden coupled with a higher immune cell repertoire resulted related to a favorable clinical outcome; a higher degree of genomic instability was present in responding patients with epithelioid mesothelioma; patients carrying germline alterations in cancer-predisposing genes, such as those involved in DNA repair, resulted more likely to be long-term survivors. Therefore, a phase III study (the PrE0506/DREAM3R trial) is now enrolling patients with unresectable and treatment-naïve MPM to compare standard platinum-based chemotherapy ± durvalumab (NCT04334759).

The phase II-III IND.227 trial is comparing cisplatin-pemetrexed ± pembrolizumab in unresectable mesothelioma patients (NCT02784171). The Beat-meso trial, a randomized phase III study, is comparing the triplet therapy of carboplatin, pemetrexed, and bevacizumab versus the quadruple therapy of carboplatin, pemetrexed, bevacizumab, and atezolizumab in 320 mesothelioma patients (NCT03762018). Considering the detailed biomarker studies planned in these trials, their results should probably guide patient selection for different therapeutic strategies.

In conclusion, the immunotherapy revolution has improved the survival outcomes of patients with a broad range of cancers, mesothelioma included. In fact, starting from the data of the randomized Checkmate-743 phase III trial, the combination ipilimumab/nivolumab gained FDA and EMA approval as first-line therapy for unselected patients with unresectable MM, giving for the first time after two decades a new option of care instead of (or in addition to) platinum-pemetrexed based chemotherapy.

## Predictive biomarkers

In order to personalize the treatments and avoid unnecessary toxicity, the main issue about the use of ICIs in MM is the needing for biological or molecular features usable as reliable biomarkers to predict which patients are more likely responders to immunotherapy. **Table 2** shows the main predictive biomarkers under evaluation in MM.

**Table 2:** Main predictive biomarkers under evaluation in MM.

	Rationale	Limits
Histology	• Known prognostic role for MPM: non-epithelioid histology considered as a predictor of poor survival (22, 23). • Evidence of better response to platinum and pemetrexed chemotherapy in epithelioid tumors (24, 25).	• Data about response according to histology are mostly not reported or too small to draw definitive conclusions (6, 8, 11, 13, 18, 19, 26). • Variable percentage of epithelioid differentiation in biphasic form, without a clear threshold value (27).
PD-L1 espression	• Known prognostic role for MPM: higher levels of expression apparently associated to poorer outcomes (28, 29). * Widely used as predictive biomarker for ICIs in particular in NSCLC.	• Conflicting data about correlation between higher levels and better responses to ICIs (6, 8, 13). • Availability of several immunohistochemistry assays (30). • No clear cut off value for defining PD-L1 positivity in mesothelioma (16, 30, 31). • Dynamic feature in disease history (32, 33).
TMB	• A greater value may lead to a higher immunogenic neoantigen exposure and, consequently, to a stronger immune activation and a greater benefit from ICIs (34). • Tumors related with carcinogenic exposure (like asbestos for mesothelioma) usually have a high TMB (35, 36). • A predictive value for ICIs response was observed in NSCLC and melanoma (37, 38).	• Availability of different sequencing assays (39–41). • No clear depth of sequencing to be performed (39–41). • Low average value (despite what was expected) (35, 36).
Genomic biomarker	• A better knowledge of the mechanisms underlying the MPM biology by genomic profiling techniques can help identifying patients more likely to be ICIs responders. • Specific genomic alterations can explain an higher neoantigens formation and/or the mechanisms underlying a greater or poorer immune activation (7, 40–54)	• Limited data about potentially useful specific genomic alterations. • Needing of time, funds and specially trained personnel to perform genomic sequencing techniques. • Not enough data to hypothesize an application in clinical practice outside of clinical trials.
TME	• The only biomarker to evaluate the immune cells infiltrating the tumor microenvironment rather than the tumor cells alone (35, 46). • Probable correlation between TME characteristics, survival and better or poorer immune activation when ICIs are administered (7, 35, 46, 49, 55–79)	• No commercially-available standardized gene panels to evaluate tumor immune microenvironment. • Not enough data to hypothesize an application in clinical practice outside of clinical trials.
Other immune checkpoint molecules	• LAG-3, is a receptor expressed on activated T cells and suppress their activation and expansion (80, 81). • TIM-3 is express on immune cells (CD8 and CD4 T cells, NKs, macrophages, DCs), it inhibits Th1 response and stimulates Tregs activation. Low levels of TIM-3 seem to correlate with improved OS in MPM patients treated with anti-CTLA4 (49, 80–82). • VISTA inhibits T cells proliferation and activation; it seems to be highly expressed in MPM rather than in other solid tumors (47, 49).	• Limited and early data, currently not sufficient to hypothesize an application in clinical practice.

MPM, Malignant Pleural Mesothelioma; PD-L1, Programmed death-ligand 1; ICIs, Immune Checkpoint Inhibitors; NSCLC, non-small cell lung cancer; TMB, Tumor Mutational Burden; TME, Tumor Microenvironment; LAG-3, Lymphocyte Activation Gene-3; TIM-3, T-cell immunoglobulin and mucin containing protein 3; VISTA, V-domain Ig suppressor of T cell activation; CTLA4, Cytotoxic T-Lymphocyte Antigen 4; NKs, Natural Killers; DCs, Dendritic Cells; Th1, T Helper 1; Tregs, T Regulatory Cells.

### Histology

Histological subtype in MPM has been widely recognized as a prognostic factor, with non-epithelioid histology considered as a predictor of poor survival in two main prognostic scores (EORTC and CALGB) (22, 23). In fact, a longer median survival has been seen in epithelioid tumors compared with non-epithelioid ones. Moreover, a better response to platinum and pemetrexed chemotherapy has been observed for epithelioid tumors (24, 25). On the other hand, it is not clear if histology can be used as an item predictive of immunotherapy response. Due to the small sample size and the low percentage of patients with non-epithelioid tumors, in several phase I and II trials testing ICIs, data about response according to histology was not reported or was too small to draw definitive conclusions (11, 18, 19).

Cedres et al. evaluated, in a retrospective cohort of 189 patients, systemic therapy outcomes according to histology (26). The study was focused on chemotherapy, confirming better results in terms of OS and PFS in epithelioid than in non-epithelioid tumors in first line setting (26.7 *vs* 15.0 months for OS, p<0.001, respectively; 4.8 *vs* 3.6 months for PFS, p=0.03, respectively). Moreover, an analysis of 27 patients receiving immunotherapy in second or subsequent lines showed a statistically significant difference in OS in favour of epithelioid histology compared with non-epithelioid one (28.3 *vs* 13.8 months, p=0.01), while no statistically significant difference was observed for PFS (2.7 months in epithelioid subtype *vs* 3 months in non-epithelioid one, p=0.43).

In a similar way, in the PROMISE-meso trial, considering the pembrolizumab arm, non-epithelioid histology showed poorer PFS and OS than the epithelioid histology (HR 1.76 and 1.54, respectively), although these data were not statistically significant (95% CI 0.58–5.33 and 0.49–4.83, respectively), probably for the small sample size (on 73 patients receiving immunotherapy, only 7 (9.6%) had a non-epithelioid histology) (13).

Moreover, in the CONFIRM trial, evaluating nivolumab versus placebo in pre-treated patients, a subgroup analysis reported a significant improvement for PFS and OS with nivolumab in epithelioid group (for PFS HR=0.64 (95% CI 0.50–0.83) and for OS HR=0.67 (95% CI 0.50–0.91)) but not in non-epithelioid one (for PFS HR=0.77 (95% CI 0.37–1.60) and for OS HR=0.79 (95% CI 0·35–1·80)) (8).

It is also interesting to consider that, in non-epithelioid mesothelioma, biphasic form can display a variable percentage of epithelioid differentiation and Vigneswaran et al. found this percentage as an independent predictor of survival (27).

Important data about the role of histology has been reported in CheckMate-743 study. This large phase III trial compared the combination of ipilimumab and nivolumab versus chemotherapy with cisplatin and pemetrexed with OS as primary endpoint in all patient. A stratification by histology (epithelioid versus non-epithelioid) was pre-planned. Among 303 patients receiving immunotherapy, histology was epithelioid in 229 patients (76%) and non-epithelioid in 74 patients (24%). Immunotherapy showed a greater benefit over chemotherapy in non-epithelioid histology than epithelioid. In particular, a median OS of 18.1 months in immunotherapy arm and 8.8 months in chemotherapy arm (HR 0.46, 95% CI 0.31–0.68) were observed for non-epithelioid histology compared to 18.7 months and 16.5 months for epithelioid histology (HR 0.86, 95% CI 0.69–1.08), respectively (6). Although the greater OS benefit in non-epithelioid group seems to be mostly related to a poor performance of chemotherapy and the trial was not specifically designed to identify a difference according to the histological subtype, also considering the consistent sample size, these findings could suggest histology as a potential biomarker in therapeutic choice, with non-epithelioid tumors having a particular benefit from immunotherapy rather than chemotherapy.

### Programmed death-ligand

Human PD-1 is a membrane protein belonging to the CD28 family and normally expressed by immune cells (T and B cells, macrophages and dendritic cells). It is involved, by interacting with its ligand PD‐L1, in negative regulation of immunity. PD-1 can be also expressed in TILs and, on the other hand, tumor cells can express PD‐L1 in different percentage, contributing to the inhibition of CD4+ and CD8+ T-cell activation and to the apoptosis of antigen-specific T-cell clones (83, 84).

The PD-L1 expression, evaluated by immunohistochemistry, seems to have a negative prognostic role in several solid tumors (85–87). Around 20-50% of MPM express PD-L1 (considering positivity of cells =>1%) and a higher level of PD-L1 expression is apparently associated to poorer outcomes and most likely observed in sarcomatoid tumors (28, 29).

However, the predictive role of PD-L1 for ICIs response in mesothelioma is not clear. In fact, since the first phase 1 and 2 studies, conflicting results have been found about a higher response rate in PD-L1 positive tumors treated with ICIs compared with negative ones.

For example, in phase 1b KEYNOTE-028 trial, enrolling only PD-L1-positive pretreated patients with MPM (with positivity defined as immunohistochemistry expression in at least 1% of tumor cells) to receive pembrolizumab, promising results in terms of durability and efficacy of response were observed (11). These data seemed to be confirmed in a phase 2 single-arm trial testing pembrolizumab in previously treated patients not selected for PD-L1 expression, showing a greater ORR in PD-L1 positive than in negative patients (26-31% *vs* 7% respectively) (88). However, no statistically significant difference in terms of response was observed in patients with MPM expressing PD-L1 compared to patients with MPM negative for PD-L1 expression in the KEYNOTE-158 trial (12). Also in the NivoMes trial, nivolumab showed no differences in terms of DCR, PFS and OS by stratifying patients enrolled according to PD-L1 status (14). On the contrary, in the MERIT trial, testing nivolumab in a similar pretreated population, an interesting trend (not statistically significant) in favor of PD-L1 positivity compared to PD-L1 negativity was reported in terms of ORR (40% (95% CI 21.9–61.3) *vs* 8% (95% CI 1.5–35.4); PFS (7.2 months *vs* 2.9 months; p=0.4490), and OS (17.3 months *vs* 11.6 months; p=0.2021) (15, 89). Similarly, in the INITIATE trial evaluating nivolumab and ipilimumab, a *post-hoc* analysis about the disease response at 12 weeks and the duration of response for more than 6 months according to PD-L1 status suggested a greater benefit in PD-L1 positive tumors compared to negative ones (RR at 12 weeks 47% *vs* 16% (p 0.018) and DOR > 6 months 73% *vs* 32% (p=0.037), respectively) (18). The MAPS2 trial, testing nivolumab and nivolumab plus ipilimumab in a non-comparative design, reported an advantage in terms of ORR but not in terms of 12-week DCR for patients with PD-L1 positive tumors (19).

Similarly, the predictive value of PD-L1 in terms of response to ICIs therapy remain controversial also in the larger phase 3 trials.

In particular, in the CONFIRM and PROMISE-Meso trials, PD-L1 expression ≥1% was not related to either PFS or OS (8, 13), while in the CheckMate-743 trial the PD-L1 positivity seemed to predict better outcomes with nivolumab plus ipilimumab over chemotherapy (6). Nevertheless, it should be noted that in this last trial the difference observed in terms of survival benefit was related to a poorer efficacy of chemotherapy in PD-L1 positive patients compared to negative ones (median OS 15.4 months and 16.6 months, respectively), while median OS with nivolumab plus ipilimumab was similar in the two groups (PD-L1 ≥1% group: 18 months; PD-L1 <1% group: 17.3 months). Moreover, in the CheckMate-743 trial, the PD-L1 status was not a stratification factor, so this datum is purely descriptive and a potential imbalance in positive and negative group could not be excluded, precluding firm conclusions (7).

Several confounding factors complicate the evaluations about the role of PD-L1 expression as a predictive biomarker.

First of all, the use of different immunohistochemistry assays and its application on tumor cells only (tumor proportion score, TPS) or both on tumor and infiltrate immune cells (combined positive score, CPS) can lead to different positivity scores and to not comparable findings between various studies (30). The majority of trials evaluating ICIs activity in MM have evaluated the PD-L1 expression only on tumor cells. However, the role of the tumor immune microenvironment in the biology of MM is known. In particular, the abundance of the tumor-associated macrophages (TAMs), which are the key inflammatory cells with a potent immunosuppressive activity, suggests a potential key role of the myelomoncytic cells in the immunosuppression in MM and in the activity of PD-1 targeting antibodies. Therefore, the valuation of PD-L1 expression not only on tumor cells but also on the tumor microenvironment cells could be more informative about prediction of response to ICIs. Nonetheless, no association with PD-L1 status (measured by both TPS and CPS) was observed for PFS and OS in the DREAM trial (20). Moreover, also if a threshold of 1% is usually been used, a clear cut off for defining PD-L1 positivity in mesothelioma has not been identified yet. In phase 1b JAVELIN trial, it was tried to evaluate avelumab anti-tumor activity according to two different cut-off values for defining PD-L1 positivity on tumor cells (≥1% and ≥5%): a similar benefit for both the threshold values in ORR, PFS and OS was observed, without better results by increasing the threshold value (16). Continuing on this topic, it is not even clear whether the peritoneal mesothelioma should be distinguished from the pleural form: the former is rarer than the latter, but it seems to express higher PD-L1 levels so has not been established if a different threshold for PD-L1 expression should be used and the very small number of cases makes this assessment difficult (30, 31). Furthermore, PD-L1 expression seems to be a dynamic feature in disease history, so an evaluation at diagnosis may not be consistent with PD-L1 status after one or more lines of treatments (32, 33).

Another open issue is the role of PD-L1 expression when ICIs are combined with other drugs, in particular with chemotherapy. For example, no association with PD-L1 status (measured by both TPS and CPS) was observed for PFS and OS in the DREAM trial (20). Results of larger ongoing phase 3 trials testing chemoimmunotherapy (DREAM3R, IND227-IFCT1901 and BEAT-meso) will probably help to clarify this issue (NCT04334759, NCT02784171, NCT03762018).

On the basis of data from these studies, the predictive value of response of PD-L1 to ICIs in patients with MM still remains weak and uncertain. However, the feeling is that tumors with a higher positivity for PD-L1 present a higher probability to benefit from immunotherapy. In example, in patients with epithelioid MM, who achieved apparently similar outcomes on combination ICIs and chemotherapy, the PD-L1 expression could be a useful marker to discern treatment selection. Certainly, a deeper study of biological characteristics of these responsive patients with a tumoral PD-L1 positivity and the identification of a standardized method to define the positivity of PD-L1 in MM (type of assay, type of cells to evaluate, threshold of positivity) may help us to better clarify the predictive value of PD-L1.

### Tumor mutational burden

The TMB is defined by the number of mutations per megabase of sequenced tumor DNA and it is considered a potential predictive biomarker of response to ICIs also in MM (90). In fact, a greater number of somatic mutations identified in tumor cells may lead to a higher immunogenic neoantigen exposure and, consequently, to a stronger immune activation which could benefit from therapy with ICIs (34). Currently, there are different sequencing assays for TMB evaluation and the depth of sequencing to be performed is not yet established. As for PD-L1, the lack of standardization of the method for determining the TMB complicates any comparison between different trials and cancer types. However, a predictive value of TMB for ICIs response was observed in patients with non-small cell lung cancer (NSCLC) and melanoma (37, 38). On June 2020, the FDA approved the use of pembrolizumab for advanced solid tumors, previously treated or without any valid alternative treatment option, with a mutational burden of at least 10 mutations per megabase, determined by an FDA-approved test (91).

Mesotheliomas usually appear to have a low average TMB of around 2 mutations per megabase, which is an unexpected finding, because tumors related with carcinogenic exposure (like asbestos for mesothelioma) usually have a high TMB, as seen in particular in NSCLC and melanoma (35, 36).

In the KEYNOTE-158 trial, a prospective exploratory analysis was planned to investigate the relation between tissue-TMB (evaluated by using the FoundationOne CDx assay) and clinical outcomes with pembrolizumab monotherapy in ten different solid tumor types, including mesothelioma (cohort H). 790 patients with evaluable tissue-TMB scores were included in the analysis and 102 of them had TMB-high status (threshold defined at ≥10 mutations per megabase). Across all tumors, an advantage in ORR was found in TMB-high group compared to non-TMB-high one (29% *vs* 6% respectively). However, considering mesothelioma cohort, on 85 evaluable cases only 1 was TMB-high and a disease response was reported in 9 of 84 TMB-low patients; notably, the same median tissue-TMB score was observed both in responders and non-responders to pembrolizumab (1.26 mutations per megabase) (92).

An exploratory analysis regarding TMB was performed also in Checkmate-743 trial. The TMB evalutation was feasible in 53% of patients treated with nivolumab plus ipilimumab and in 45% of patients treated with chemotherapy arm, with the evidence of a median low value (1.75 mut/Mb). However, in this analysis, a higher mutational burden was not correlated to a higher OS in either the immunotherapy or chemotherapy arm.

Based on the results available to date, TMB seems not particularly promising to predict ICIs efficacy in mesothelioma. Moreover, it should be considered that, for its evaluation, next-generation sequencing is traditionally used to identify single nucleotide variation and this technique seems not able to identify the complex chromosomal rearrangements with neo-antigenic potential observed in mesothelioma (39–41).

### Genomic biomarkers

Despite the low TMB of MPM and the lack of predictivity of PD-L1 to immunotherapy response, several analyses of the genomic landscape of MM suggested interesting signs to understand the basis for a response to ICIs. Chromosomal rearrangements such as insertions, deletions, and chromosomal translocations are frequently found in MM. Mansfield et al. observed wide inter- and intra-chromosomal rearrangements in the form of chromo-anagenesis, such as chromoplexy or chromothripsis, in 86% of MM samples analyzed (40). Chromothripsis is a pattern of different chromosomal rearrangements resulting from multiple double-strand breaks and reassembly of a long segment or an entire chromosome (42). Chromothripsis has been associated with a worse prognosis in MM patients (40). However, this structural chromosomal variant, also called tumor junction burden, is associated with potential neoantigens formation that facilitates intra-tumural expansion of T-cell clones, suggesting that chromothripsis could have a role in the response to immunotherapy (40). Kosari et al., studied the relationship between tumor junction burden and OS in MM patients treated with nivolumab or nivolumab plus ipilimumab relapsed after first line chemotherapy. Even if tumor junction burden didn’t directly demonstrate a predictive role, its strong correlation with “antigen presentation and antigen processing” (APP) gene signatures predicted longer OS. In particular, considering that the impact of tumor junction burdens seemed to be modulated by APP, Kosari and collegues hypothesized that the neo-antingenic potential of chromosomal rearrangements was dependent on the capability of cancer cells to present neoantigens to the immune system. Therefore, to test whether there was an interaction between APP gene sets and tumor junction burdens that affected outcomes, they selected 12 APP gene sets from the Gene Ontology Biological Processes data set in the Molecular Signature Database and calculated their enrichment scores. Using these scores to test for interactions between APP gene sets and junction burdens on survival they found significant interactions with six APP gene sets. With these six APP gene sets, the HRs representing associations between tumor junction burdens and OS favored patients with high APP scores (all HRs < 1) more so than patients with low APP scores (all HRs > 1). Moreover, patients with a low APP gene expression and a high tumor junction burden showed a worse prognosis compared to patients with high APP score and high tumor junction burden when treated with ICIs (42). This is in line with the concept that ICIs need neoantigens presentation on cancer cells to activate cytotoxic T-cell antitumor response (42). Therefore, if prospectively confirmed, this interaction signature between the tumor junction burdens and APP gene sets could represent a potential biomarker for immunotherapies patients’ selection in clinical practice.

In general, MM is driven by commonly occurring somatic copy-number alterations at the genomic level. These alterations involve loss of a small number of tumor suppressor genes such as BRCA1-associated protein 1 (BAP1) (located in 3p21) and CDKN2A (located in 9p21), meanwhile oncogenic gain-of-function alterations are rare. The genomic structural variants, characterizing these genes loss of function, are often in the form of chromothripsis (43–47).

BAP1 (BRCA1-associated protein 1 carboxy-terminal hydrolase) is a tumor suppressor gene that modulates gene expression regulating histone H2A activity. It is also implicated in the regulation of apoptosis and DNA replication and repair (48, 49). BAP1 is the most common mutated gene in MM, with its alterations (somatic mutations and deletions) found in ~55% of cases (44–48). In particular, BAP1 mutations are characteristic of epithelioid MM more than of other subtypes (49). BAP1 alterations are found both in the germline and the somatic setting. The heterozygous germline alterations have an autosomal dominant hereditary pattern and people inheriting these alterations have a higher risk of developing MM (especially after asbestos exposure), melanoma, clear-cell renal cell carcinoma and cholangiocarcinoma (43, 44, 49). Forde et al. in the PrE0505 trial, testing the efficacy of durvalumab plus chemotherapy in MM first line setting, demonstrated that BAP1 germline mutations were associated with a significantly prolonged survival after chemo-immunotherapy. Moreover, other MPM associated germline loss-of-function mutations (MLH1, MLH3, BRCA1, BRCA2 and BLM), in particular those associated with DNA damage repair mechanisms, have been linked to a longer OS (p=0.05 in all MMs analysed and p=0.032 in epithelioid MMs) (44). A possible explanation for this phenomenon is that the tumor immune microenvironment in BAP1 muted gene is more inflammatory. In fact, Forde et al. demonstrated that BAP1 null MM had an increased CD8+ T cell infiltration and higher levels of granzyme B transcripts, indicating an active cytotoxic tumor immune microenvironment (TIME), suggesting that MMs with BAP1 loss may be more responsive to immunotherapy (35, 44). Hmeljak et al. demonstrated that BAP1 loss of function mutations are associated with an upregulation of IRF8. IRF is a transcription factor that regulates interferon signalling and dendritic cells differentiation (particularly CD103+), the latter importantly involved cytotoxic T cells’ stimulation in the tumor immune microenvironment (TIME). This finding supports BAP1’s role in influencing the TIME (47).

One of the most frequent copy-number mutation in MM is 9p21 deletion, which contains CDK2NA and MTAP (its adjacent gene). This alteration is associated with worse prognosis and with primary resistance to immune checkpoint therapy (45, 46, 50). Han et al., in pan-cancer analysis of The Cancer Genome Atlas (TCGA) data of eight ICIs trials, demonstrated that 9p21 deletion is associated with a “cold” tumor microenvironment characterized by diminished T, B, and NK cells’ infiltration, reduced immune cell activation, lower PD-L1 expression levels and a stronger immunosuppressive signalling (50). Considering that almost 50% of the TCGA MM samples presents 9p21 loss, this mechanism represents an important explanation of ICIs’ resistance in MPM (35). Moreover, in an extensive genome analysis of MPM, Nastase et al. (45) revealed that CDKN2A loss on 9p21.3 was frequently associated with the deletion of the near located Type I Interferon (IFN) genes (found delated in 52% of samples). IFNs induce a pro-inflammatory status in the tumor microenvironment. In melanoma, IFN loss of function has been related to a reduced response to CTLA4 inhibition. Even if Nastase et al. did not found a statistically significant difference in OS in patients with CDKN2A and IFN type I co-deletion, this genomic alteration may have a role in MPM cells immune escape.

Zhang et al., using an exome sequencing approach of MM samples, identified 5 genomic clusters, characterized by a temporally ordered tumorigenesis and bearing a prognostic value (48). These evolutionary clusters ranged from low (cluster 1) to high (cluster 5) complexity. The phylogenetic evolution analysis showed that loss of BAP1/−3p21, FBXW7/-chr4 and 9p21.3 were always early clonal events in MM tumorigenesis, demonstrated by their presence in nearly all subclones. Instead, Hippo pathway inactivation, caused by NF2/−22q events, are mainly late events occurring only in some subclones. The loss of Hippo pathway activity is found in advanced, more aggressive MPMs and is associated with chemoresistance, suggesting its role as critical bottleneck in the tumoral evolution. The MMs’ clonal neoantigen architecture modulates the immune surveillance, and so it has the potential to be a biomarker of response to ICIs. Interestingly, evolutionary cluster C5 has the highest degree of repeated early clonal alterations (and so of neoantigen burden), the worst OS, the highest CD8 T lymphocyte infiltration, and, at the same time, has the inferior Treg cell infiltration rate. Of note, in Zhang et al.’s MEDUSA cohort, C5 was found only in the epithelioid subtype, thus demonstrating a subset of patients with a worse survival in epithelioid MPMs. For these reasons cluster C5 could be a potential predictor of response to immunotherapies (48).

In the PrE0505 trial Forde et al., considering that DNA breaks are common in MM, assessed chromosomal instability quantifying copy number breakpoints in the samples’ genome. The authors identified these alterations more frequently in epithelioid MPM of patients with an OS of 12 or more months (p=0.053). This supports the hypothesis that DNA breaks, and their potential of neoantigen formation, are a positive prognostic biomarker in MPM and a possible predictor of response to ICIs (44).

In this trial it was also demonstrated that MMs characterized by a high variability in T-cell receptor (TCR) clonality had an increased survival with chemo-immunotherapy (OS>21 months). The authors also showed that an increased immunogenic mutations burden in major histocompatibility complex (MHC) class I and MHC class II was significantly associated with a better response to durvalumab plus chemotherapy (p=0.064 and p=0.023, respectively), especially in the epithelioid subgroup. Moreover, they demonstrated that a higher human leukocyte antigen (HLA)-B locus divergence was linked to an improved radiological response to chemoimmunotherapy, in particular in epithelioid MMs (p=0.06 and p=0.003, respectively) (44). This evidence is in line with HLA class I allele divergence hypothesis, suggesting a better tumor immune response when there is a high HLA class I functional variability. HLA loss of heterozygosity (LOH), *via* immunoediting, reduces antigen presentation by the MHC, thus consenting tumor escape from CD8-T cells immune response. HLA LOH is a late event in MPM clonal evolution. C5 cluster MPMs are the most frequently interested by HLA LOH, another potential explanation of a *de novo* or acquired ICIs resistance in MM (48, 51).

Forde et al., in the PrE0505 study, showed that MMs responding to chemo-immunotherapy had a higher frequency of non-synonymous missense mutations and clonal mutations than those not responding (p=0.086 and p=0.072), in particular in the epithelioid subgroup (p=0.051 and p=0.025, respectively). In line with these data, the authors demonstrated a strong correlation between APOBEC mutational signature, underpinning subclonal mutagenesis, and non-responsive epithelioid MMs (p=0.031). They hypothesized that a high subclonal mutation burden, in part caused by an altered function of the APOBEC enzymes, could permit tumor immune evasion (44).

Inflammatory gene signature scores have demonstrated a positive predictive role to immunotherapy in other cancer types (melanoma, gastroesophageal cancer and advanced hepatocellular carcinoma) (52–54). In the Checkmate-743 exploratory analysis, the expression of CD8A, STAT1, LAG3, and CD274 (PD-L1) was quantified using RNA sequencing. This analysis demonstrated that a high four-gene inflammatory signature score was associated with an OS benefit in the nivolumab plus ipilimumab arm (mOS 21.8 months versus 16.8 months in patients with low score). In the chemotherapy arm no correlation between inflammatory gene signature score and response was identified. Inflammatory signature score, could, thus, be considered a positive predictive biomarker of response to immunotherapy (7).

### Tumor immune microenvironment

The TIME consists mainly of tumor associated macrophages (TAM), myeloid-derived suppressor cells (MDSC), CD4- CD8- T cells, B lymphocytes, NK cells, DCs, stromal and endothelial cells. TAM are the most represented immune cell type in TIME of MM (~20–40% of the immune infiltrate). Among TAM, M2 macrophages are predominant, indicating therefore an immunosuppressive phenotype (35, 46). Ollila et al. in a study evaluating immune cells infiltrating the tumor microenvironment of MM and their relationship with survival, demonstrated that M2 macrophages, mediators of tissue remodelling (CD163+ pSTAT1− HLA-DRA1−), are associated with low OS whereas proinflammatory M1 macrophages (CD68+ pSTAT1+ HLA-DRA1+) have a positive correlation with survival (55). The M2 macrophages seems to have a role in stimulating tumor proliferation and invasiveness. Moreover, the M2 macrophages are potent cytotoxic T lymphocyte (CTL) suppressors, and often express PD-​L1, thus favouring tumor immune escape (35). Creaney et al. demonstrated a high expression of CCL2, TGFβ1, MMP14 and MMP2 (MMP: Matrix metalloproteases) chemokines in the MM’s TIME. These chemokines, seemingly secreted by tumor cells, were correlated with M2 macrophages infiltration, suggesting that they may contribute to an immune suppressive environment. Transforming growth factor beta (TGFβ1) is involved in M2-like macrophage differentiation, and its expression correlates with disease stage, tumor volume and shorter survival (46, 49). Monocyte chemoattractant protein-1 (CCL2) is an important TAM-associated chemokine, responsible for T cells, macrophages, and dendritic cells infiltration in the tumor microenvironment. Moreover, it has been noted that CCL2 levels are related to tumor stage, suggesting that macrophages play an important role in cancer progression (49).

Various studies evaluated the role of TAMs as negative predictive biomarker of response to immunotherapy. Indeed, TAMs can bind the Fc-domain glicans of anti-PD-1 antibodies on PD-1+ T cells with TAM’s Fcγ-receptor, thus reducing T cells exposure to anti-PD-1 antibodies (56–59). TAM predictive role in neoplastic patients treated with ICI has been shown in various cancer types, as NSCLC, melanoma, glioblastoma and urothelial carcinoma (60–64). Further studies should be conducted to demonstrate a predictive role of TAMs also in MM.

In MM T-lymphocytes represent ~30% of the TIME, comprising CD4+ T cells and CD4+/FOXP3+ Tregs (1–50%) and CD8+ CTLs (5–15%) (65). Mankor et al. conducted a retrospective immune-monitoring analysis on peripheral blood samples of MM patients treated with either nivolumab (NivoMes trial) or nivolumab plus ipilimumab (INITIATE trial), to assess the predictive role of tumor infiltrating lymphocytes (14, 18, 66). The authors demonstrated, in patients treated with aPD-1/aCTLA-4 combination, a relationship between response and a low rate of naive CD8 T cells (CD45RA+ CCR7+), a high rate of pre-treatment “terminally differentiated effector memory T cells” (TEMRA; CD8 T cells CD45RA+ CCR7+) and high frequency of pre-treatment TEMRA expressed Granzyme-B and Interferon-γ cytokines. Moreover, in patients treated with the combination immunotherapy, increased memory T-cells proliferation and CD4- CD8- T cells activation were shown. These proliferation and activation were not related to response, suggesting that nivolumab plus ipilimumab induced a non-tumor specific T cells response. Only patients with pre-ipilimumab plus nivolumab high TEMRA rate had a benefit from treatment, indicating that TEMRAs mediate a tumor specific response. In nivolumab monotherapy patients these correlations were not found indicating that only an anti-CTLA4/anti-PD-1 treatment can reactivate TEMRAs. To conclude peripheral blood TEMRAs could be used as a predictive biomarker of response to combination immunotherapy (66).

Identifying the relations existing between MM immune cells and prognosis may be the first step towards the identification of predictive therapeutic biomarkers in this setting.

In 2017 Chee et al. (67) evaluated the prognostic role of infiltrating T-cells (CD8+, FOXP3+, CD4+, CD45RO+, CD3+), B-cells (CD20+), neutrophils (NP57+), NK cells (CD56+) and macrophages (CD68+) in MM’s patients. The authors observed that FOXP3+ CD4+ Tregs are related to a worse survival, in line with their inhibitory activity on effector and helper T-cells. FOXP3+CD4+ Treg cells account for 2.8% of the total CD4+ lymphocytes in MM (68). Further, in epithelioid MMs, a CD4+/CD8+ ratio >1 and a high frequency of CD4+ T cells were associated with an improved survival, consistent with CD4+ T lymphocytes’ role in the stimulation of CD8+ TILS and B-cells against cancer cells. This study demonstrated that a high CD8+ T-cells infiltration in MPM’s TIME was not associated with an improved survival, as confirmed in a recent RNA-seq analysis of TIME by Creaney et al. (46).

The B cells represent 4% of TIME and have a central role in the immune crosstalk, acting as both positive and negative regulators of cancer. Their role as positive tumor regulators has been associated with B cells that express “signal transducer and activator of transcription 3” (STAT3), that contributes to a proangiogenic environment thus promoting tumor growth (67, 69). Tumor-associated B-cells’ role as negative regulator could be explained by their ability to act as antigen presenting cells inducing CD4+ T cells activation, differentiation, and polarization in Th1 and Th2 subtypes. A high density of CD20+ B lymphocytes in the TIME is associated with an increased survival in patients affected by epithelioid MM (49, 69). This evidence is notable considering that a high B cell infiltrate was found in approximately 50% of MMs in Patil et al.’s retrospective analysis (70).

B cells play also a role in the formation of tertiary lymphoid structures (TLS). TLS are organized ectopic lymphoid aggregates that arise in chronically inflamed environments like autoimmune diseases, chronic infection, and cancer (69, 71). TLSs have been identified in different cancer types and have been associated with a better prognosis and response to immunotherapy. So not only immune cell infiltrating the TME, but also their organisation in TLS is important for anti-tumor immune response (71–75).

TLSs are associated with the local immune response, the ability of germinal centres formation and the lymphocytes’ recruitment. Tumors harbouring TLSs in their microenvironment are characterized by an increased immune infiltration. TLSs do not have a capsule, so immune cells resident in them could be directly exposed to macromolecules from the TME, thus driving intratumoral immune response. B cells in TLS can produce antibodies that tie antigen expressing tumor cells inducing subsequent opsonisation, complement-dependent lysis, or antibody mediated cytotoxicity. Moreover, tumors harbouring TLSs and an important CD8+ T cells infiltration have a better prognosis than those characterized only by CD8+ T cells infiltration, suggesting a better immune response quality in tumors with the TLSs. Immunotherapy promotes TLS formation and activity and this can explain the possible role of TLS and B cells infiltration as positive predictive biomarker of response to ICIs in different cancer types (69, 71, 76, 77).

Based on these evidence Mannarino et al. conducted a retrospective multicenter cohort study of MPM patients never treated with chemotherapy in order to identify TIME features potentially predictive of patients’ outcome. The authors demonstrated that epithelioid MM patients with long OS (>36 months) were characterized by an inflammatory background with a higher expression of B-cells (CD20+) and prevalence of TLS formations compared to epithelioid MM patients with short OS (<12 months), which showed a higher frequency of neutrophils and M2 macrophages (p = 0.025) (69). Therefore, B cells showed a negative impact in cancer development in this study. As said before, a possible explanation is B cells’ role in antigen presentation and cytotoxic antitumor T cells activation. In particular MM, even if characterized by a low TMB, is often interested by chromothripsis, and, thus, tumor junction burden with the potential of neoantigens formation. These neoantigenes could be at the origin of B-cells mediated antitumor immunity (69).

Chee et al. demonstrated that a low rate of NP57+ neutrophils in the TIME of epithelioid MPM was associated with better OS. This finding is consistent with the hypothesis that tumor-associated neutrophils can have a facilitating role in tumorigenesis by promoting angiogenesis and facilitating the development of pro-invasive and pro-metastatic TIME (67).

Ollila et al. studied the correlation between tumor infiltrating immune cells and MM’s prognosis thanks to TIME high-resolution deep profiling (55). This analysis demonstrated that granzyme B/CD11c positivity was significantly associated with a better OS. Myeloid-derived cells express CD11c. Thanks to immunohistochemistry the authors identified these prognostic favourable CD11c cells to be DCs. DCs have a fundamental role in antigen presentation and consequently in immune activation in TIME of various cancers. Granzyme B is produced by various immune cells, including DC. In conclusion granzyme B and CD11c could be used as prognostic biomarkers and further studies should be conducted to evaluate their role in predicting immunotherapy response in MPM patients.

Ollila et al. also demonstrated that myeloid derived suppressor cells (MDSCs) in the TME are related with a shorter OS (55). The MDSC are abnormal granulocytes that develop in pathological conditions. They have a prevalence of less than 10% in the TIME. The MDSCs favour tumorigenesis and cancer progression *via* inhibition of Tcells activation and proliferation, and promoting TIME reshaping, epithelial to mesenchymal transition and angiogenesis (49).

Lung immune prognostic index (LIPI) score is a prognostic factor in different cancer types, including non-small cells lung cancer (78, 79). It evaluates the presence of a neutrophils/leukocytes minus neutrophils ratio greater than 3 and a lactate dehydrogenase (LDH) level greater than upper limit of normal, thus identifying 3 groups (good, 0 factors; intermediate, 1 factor; poor, 2 factors). In an exploratory analysis of possible ICIs’ predictive biomarkers in the Checkmate-743 phase 3 trial, LIPI score seemed to have a prognostic role with an improved OS in patients with a good score than in those with an intermediate or poor score across both treatment arms. LIPI score didn’t have a predictive role for response to immunotherapy in CheckMate-743 trial (7).

Considering the central role that immunotherapy is gaining in MM, other immune checkpoint molecules besides PD-1/PD-L1, especially “lymphocyte activation gene-3” (LAG-3), “T-cell immunoglobulin and mucin containing protein 3” (TIM-3) and “V-domain Ig suppressor of T cell activation” (VISTA) represent a new interesting field of study.

VISTA is a negative checkpoint regulator that inhibits T cells proliferation and activation. It is expressed on the surface of myeloid cells, in particular on TAM. As PD-L1, VISTA can induce differentiation of naïve T cells to FoxP3+ regulatory T cells. VISTA can also play its inhibitory role on T cells both as receptor on T cells and as ligand on antigen presenting cells (47, 49, 80).

Hmeljak et al., conducting a comprehensive integrated genomic analysis of MPM samples, demonstrated high levels of VISTA mRNA in MPM, higher than in other solid tumors analysed in TCGA (47). Among MMs samples, VISTA expression reached the highest levels in the epithelioid subgroup. High VISTA expression levels have been related to an improved OS (47, 49). Considering that physiological mesothelium harbours APC properties that could be maintained in cancer, Hmeljak et al. performed an immunohistochemistry analysis of epithelioid MPM samples, normal and reactive mesothelium, in order to identify differences in VISTA expression. VISTA protein was demonstrated on infiltrating immune cells, in epithelioid MPM cells, in normal and reactive mesothelium. This evidence suggests that epithelioid MPMs, the more differentiated MPM subtype, retain APC properties, frequently lost in the other less differentiated subtypes. Another hypothesis is that VISTA expression in MPM is positively selected by immune pressure (47). In conclusion, VISTA should be further investigated as a possible predictive biomarker of response to ICIs.

TIM-3 is another immunosuppressive molecule, expressed on immune cells (CD8 and CD4 T cells, NKs, macrophages, DCs), that inhibits Th1 response and stimulates Tregs activation. TIM-3 ligand is Galectin-9. TIM-3 and Galectin-9 are frequently found in PD-L1 positive MMs (49, 80). TIM-3 is expressed on MPM cells and TILs of MPM, and particularly on NK cells (less on CD4+ and CD8+ T-cells) (81). Sottile et al. demonstrated an association between low levels of TIM-3 and improved OS in MPM patients treated with anti-CTLA4 (82). So, TIM-3 could have a role as predictive biomarker to immunotherapies in MPM.

LAG-3 is an immune checkpoint receptor, with the ability to suppress T-cells activation and expansion. It is expressed on activated T cells and has been found in pleural effusion of MPM patients and on TILs in pleural effusions. Its role on response to ICIs in MPM has still to be clarified (80, 81).

The T cell immunoglobulin and ITIM domain (TIGIT), an inhibitory immunoreceptor, recently emerged as a novel potential target for immunotherapy. As this novel immune checkpoint is largely unexplored in MM, the TIGIT blockade should be evaluated as an alternative therapeutic approach also for MM (93).

## Discussion

Recent years have witnessed significant improvements in our understanding of mesothelioma’s biology and innovative strategies are changing the range of therapeutic options. The main breakthrough has been made in the field of immunotherapy. In fact, the immunotherapy revolution has improved the survival outcomes of patients with a several types of cancers, and mesothelioma is now at the forefront. Recently, the FDA and the EMA approved the combination of ipilimumab and nivolumab as new standard of care for unselected patients with unresectable MM in the first-line setting (6). Despite these exciting results, it is still unclear which mesothelioma patients actually benefit from immunotherapy and which do not. In the Checkmate-743 trial, 28% of responsive patients to the combination ipilimumab-nivolumab, still remained responsive after 36 months. On the other hand, 18% of patients treated with immunotherapy resulted primary refractories compared to 5% of patients treated with chemotherapy. The occurrence of early progression or even hyper-progressive disease in MM patients treated with ICIs have been reported also in other studies (94). Moreover, the combination of ipilimumab and nivolumab in first line setting mainly benefits non-epithelioid patients, in part due to the fact that chemotherapy is ineffective for this histotype, whereas the same level of benefit was not observed in epitheliod patients. In second-third line of therapy, PD-1 inhibitors as monotherapy have been found to be superior to placebo in terms of OS and PFS in the CONFIRM trial, but not superior to chemotherapy (vinorelbine or gemcitabine) in the PROMISE-meso trial (8, 13). Both CONFIRM and the PROMISE-meso trials reported responses with ICIs in epithelioid patients. In particular, in the PROMISE-meso trial the ORR was 22% in the epithelioid patients treated with pembrolizumab compared to 6% in patients treated with chemotherapy. Therefore, considering these incoherent results and discrepancies with the use of ICIs for the therapeutic strategy of mesothelioma, it is crucial to identify predictive biomarkers, especially for epithelioid patients where benefit with immunotherapy is less definite.

In general, several efforts are underway to identify predictive biomarkers of response to ICIs. Unlike other cancers, the predictive value of response to ICIs of PD-L1 and TMB in patients with MPM still remains weak and uncertain. Probably, this is due to the extensive tumoral genomic heterogeneity among patients and histological differences typical of mesothelioma (12, 13).

The genomic research and the study of the TIME are testing several new potential predictive biomarkers. In particular, the inclusion of genomic approaches able to detect structural variants, and transcriptomics to evaluate antigen processing and presentation, could improve the selection of patients to immunotherapy. In an exploratory analysis of Checkmate-743 trial, the expression of CD8A, STAT1, LAG3, and CD274 (PD-L1) was quantified using RNA sequencing. A high four-gene inflammatory signature score was associated with an OS benefit in the nivolumab plus ipilimumab arm (mOS 21.8 months versus 16.8 months in patients with low score), suggesting his potential positive predictive role. However, these data require prospective validation.

The TIME of mesothelioma is very complex. Cancer-associated fibroblasts, T-cells, TAMs, and MDSC have immunosuppressive roles in mesothelioma (95). Interestingly, Mannarino and collegues demonstrated that epithelioid MM patients with long OS (>36 months) were characterized by an inflammatory background with a higher expression of B-cells (CD20+) and prevalence of TLS formations compared to epithelioid MM patients with short OS (<12 months), which showed a higher frequency of neutrophils and M2 macrophages (p = 0.025). TLSs have been identified in different cancer types and have been associated with a better response to immunotherapy. Moreover, immunotherapy seems to promote TLS formation and activity and this can explain the possible role of TLS and B cells infiltration as positive predictive biomarker of response to ICIs in different cancer types (69, 71, 76, 77).

Lastly, other immune checkpoint molecules besides PD-1/PD-L1, especially LAG-3, TIM-3 and VISTA, represent new interesting biomarkers. In particular, VISTA is expressed on the surface of myeloid cells, especially on TAM, and it is a negative checkpoint regulator that inhibits T cells proliferation and activation. Interestingly, pleural mesothelioma displays the highest expression levels of VISTA among all the cancers studied, particularly in the epithelioid subgroup. Therefore, VISTA is under investigation as a potential predictive biomarker of response to ICIs in mesothelioma and it could become one of the potential targets for overcoming immunotherapy resistance and a molecular target to improve the immune downregulation (96–98).

In conclusion, despite the recent therapeutic progress in mesothelioma, our knowledge of the factors that underpin response to ICIs is limited. The interpatient genomic heterogeneity and the evidences suggested by the immune-modulating therapies are supporting the need of biomarkers able to guide the selection of patients benefiting from specific and personalized therapeutic strategies. In fact, a deep understanding of the mechanisms associated with primary and secondary resistance to ICIs will further improve the outcomes of patients with mesothelioma.

## Author contributions

All authors listed have made a substantial, direct, and intellectual contribution to the work and approved it for publication.
